# Equitability of Depression Screening After Implementation of General Adult Screening in Primary Care

**DOI:** 10.1001/jamanetworkopen.2022.27658

**Published:** 2022-08-18

**Authors:** Maria E. Garcia, Ladson Hinton, John Neuhaus, Mitchell Feldman, Jennifer Livaudais-Toman, Leah S. Karliner

**Affiliations:** 1 Center for Aging in Diverse Communities, University of California, San Francisco, San Francisco; 2 Multiethnic Health Equity Research Center, Division of General Internal Medicine, Department of Medicine, University of California, San Francisco, San Francisco; 3 Division of General Internal Medicine, Department of Medicine, University of California, San Francisco, San Francisco; 4 Implementation Science Training Program, Department of Epidemiology and Biostatistics, University of California, San Francisco, San Francisco; 5 Department of Psychiatry and Behavioral Sciences, University of California, Davis, Davis

## Abstract

**Importance:**

Depression is a debilitating and costly medical condition that is often undertreated. Men, racial and ethnic minority individuals, older adults, and those with language barriers are at increased risk for undertreatment of depression. Disparities in screening may contribute to undertreatment.

**Objective:**

To examine depression screening rates among populations at risk for undertreatment of depression during and after rollout of general screening.

**Design, Setting, and Participants:**

This cohort study from September 1, 2017, to December 31, 2019, of electronic health record data from 52 944 adult patients at 6 University of California, San Francisco, primary care facilities assessed depression screening rates after implementation of a general screening policy. Patients were excluded if they had a baseline diagnosis of depression, bipolar disorder, schizophrenia, schizoaffective disorder, or dementia.

**Exposures:**

Screening year, including rollout (September 1, 2017, to December 31, 2017) and each subsequent calendar year (January 1 to December 31, 2018, and January 1 to December 31, 2019).

**Main Outcomes and Measures:**

Rates of depression screening performed by medical assistants using the Patient Health Questionnaire-2. Data collected included age, sex, race and ethnicity, and language preference (English vs non-English); to compare English and non-English language preference groups and also assess depression screening by race and ethnicity within the English-speaking group, a single language-race-ethnicity variable with non–English language preference and English language preference categories was created. In multivariable analyses, the likelihood of being screened was evaluated using annual logistic regression models for 2018 and 2019, examining sex, age, language-race-ethnicity, and comorbidities, with adjustment for primary care site.

**Results:**

There were 52 944 unique, eligible patients with 1 or more visits in one of the 6 primary care practices during the entire study period (59% female; mean [SD] age, 48.9 [17.6] years; 178 [0.3%] American Indian/Alaska Native, 13 241 [25.0%] English-speaking Asian, 3588 [6.8%] English-speaking Black/African American, 4744 [9.0%] English-speaking Latino/Latina/Latinx, 760 [1.4%] Pacific Islander, 22 689 [42.9%] English-speaking White, 4857 [9.0%] English-speaking other [including individuals who indicated race and ethnicity as other and individuals for whom race and ethnicity data were missing or unknown], and 2887 [5.5%] with language barriers [non–English language preference]). Depression screening increased from 40.5% at rollout (2017) to 88.8% (2019). In 2018, the likelihood of being screened decreased with increasing age (adusted odds ratio [aOR], 0.89 [95% CI, 0.82-0.98] for ages 45-54 and aOR, 0.75 [95% CI, 0.65-0.85] for ages 75 and older compared with ages 18-30); and, except for Spanish-speaking patients, patients with limited English proficiency were less likely to be screened for depression than English-speaking White patients (Chinese language preference: aOR, 0.59 [95% CI, 0.51-0.67]; other non–English language preference: aOR, 0.55 [95% CI, 0.47-0.64]). By 2019, depression screening had increased dramatically for all at-risk groups, and for most, disparities had disappeared; the odds of screening were only still significantly lower for men compared with women (aOR, 0.87 [95% CI, 0.81 to 0.93]).

**Conclusions and Relevance:**

In this cohort study in a large academic health system, full implementation of depression screening was associated with a substantial increase in screening rates among groups at risk for undertreatment of depression. In addition, depression screening disparities narrowed over time for most groups, suggesting that routine depression screening in primary care may reduce screening disparities and improve recognition and appropriate treatment of depression for all patients.

## Key Points

**Question:**

Is implementation of routine depression screening in primary care associated with improved screening rates for groups at risk for undertreatment of depression?

**Findings:**

In this cohort study of 52 944 adult patients in primary care practices in a California health system, depression screening rates increased from 40.5% in 2017 to 88.8% in 2019 after implementation of a general screening policy. Initial statistically significant screening disparities among older patients, Black/African American and other English-speaking patients, and patients with language barriers disappeared by 2019, although disparities for men did not.

**Meaning:**

These results suggest that implementation of depression screening may reduce disparities in screening and could improve recognition and appropriate treatment of depression for all patients.

## Teaser

This cohort study assesses rates of depression screening among groups at risk for undertreatment of depression after initiation of routine primary care screening for adults in a large health system.

## Introduction

Depression is a leading cause of disability in the US, second only to cardiovascular disease. Depression has been linked to poor management of and higher rates of complications from other chronic conditions,^1,2,3,4^ higher odds of functional disability,^5^ increased health care costs and services utilization,^6^ and increased cardiovascular and all-cause mortality compared with individuals without depression.^7,8,9^ While effective evidence-based treatment models exist, they improve outcomes for patients only if depression is recognized and appropriately treated.^10,11,12,13^ Primary care is often the gateway to depression treatment, with an estimated 60% of patients receiving depression care in primary care settings and 79% of all antidepressant prescriptions written by non–mental health care professionals,^14,15^ yet depression goes unrecognized in more than half of individuals presenting with depressive symptoms in primary care, and the condition is therefore left untreated.^16^ Underrecognition of presenting symptoms occurs more frequently among men, racial and ethnic minority individuals, individuals with language barriers, and older adults.^17,18,19,20^ Furthermore, these populations are less likely to receive adequate depression care and may be at higher risk for adverse outcomes as a result of undertreatment.^1,8,18,19,21,22^

While depression screening in primary care has the potential to improve depression recognition and decrease existing depression care disparities, screening rates in primary care are low.^23,24,25^ In an analysis of National Ambulatory Medical Care data from 2012 and 2013, depression screening occurred in only 4.2% of all visits, with Black patients being half as likely to be screened compared with White patients and older adults being less likely to be screened than middle-aged patients.^24^ Another study,^25^ using data from the Agency for Healthcare Research and Quality Medical Expenditure Panel Survey, found that a higher proportion (48.6%) of adults aged 35 or older reported being screened for depression in 2014 and 2015, with lower rates among men, people over 75 years old, and racial and ethnic minority patients. However, this study included individuals with known depression and those receiving treatment for depression and may not represent screening of individuals at average risk.^25^

In 2016, the US Preventive Services Task Force (USPSTF) recommended depression screening in the general adult population to diagnose depression and initiate care.^26^ While implementation of evidence-based clinical guidelines can improve patient outcomes and public health,^27^ adoption of clinical guidelines in practice is often delayed because of poor awareness of guidelines, low agreement or self-efficacy, poor communication to users of the evidence-based practice, or the presence of external barriers to following the recommendations.^28,29,30,31,32^ Furthermore, while systematic depression screening could increase case finding and treatment engagement of vulnerable populations, systems implementation may exacerbate or lead to new disparities if geared toward the racial and/or ethnic and linguistic majority or if the local context is not considered.^32,33^ Examining data from the period immediately after the USPSTF guideline, 1 study in a large integrated health system that incorporated electronic clinical decision support for systematic depression screening reported higher screening rates (59%) than had previous studies.^34^ Another study of uninsured primary care patients reported moderately higher screening rates (67%).^23^

Despite multiple previous studies demonstrating low recognition of depressive symptoms and treatment initiation among men,^17,25^ older adults,^24,25,35^ racial and ethnic minority individuals,^17,20,21,22,24,25,35,36^ and individuals with limited English proficiency,^17,20,22^ to our knowledge, no studies have evaluated the association of patient characteristics with the likelihood of being screened since the 2016 USPSTF guideline^26^ recommended screening the general adult population. Thus, the question remains whether general depression screening in clinical practice is associated with equitable screening for all population groups. The objective of this study was to investigate whether implementation of annual depression screening of adults in a large academic US health system in response to the USPSTF 2016 guideline^26^ was associated with equitable screening rates for groups at risk for underrecognition and undertreatment of depression.

## Methods

This study was approved by the University of California, San Francisco (UCSF) Institutional Review Board with a waiver of consent because the only record linking the participant and the study would be the consent form (presenting a potential for breach of confidentiality) and there is minimal risk to participants. The study followed the Strengthening the Reporting of Observational Studies in Epidemiology (STROBE) reporting guideline for cohort studies.^37^

### Setting

University of California, San Francisco, Health (UCSF Health) is an academic health system with more than 2 million outpatient visits per year. Seven adult primary care practices, with 207 clinicians, serve approximately 68 150 empaneled adults. Most patients are insured, with a range of payors, including private insurers (63.3%), Medicaid (10.3%), and Medicare (18.5%). The patient population is 59.1% female, 28.2% Asian, 10.0% Latino/Latina/Latinx, and 7% Black/African American, and 6.3% of patients have limited English proficiency.

### Implementation of Depression Screening

A pilot test of adult depression screening was first conducted in 1 practice starting in September 2017. Screening was to be performed by medical assistants; medical assistant workflows for screening were established, and a screening template (flow sheet) was incorporated into the electronic health record (EHR). Once the workflow and flow sheet were developed, all UCSF Health primary care practices implemented annual depression screening on October 1, 2017. Prior to 2017, individual clinicians may have included screening in their notes as free text or by scanning screening questionnaires (which cannot be searched) into the clinical chart; however, this information was not systematically captured in the local EHR (Epic Systems Corp).

The health system used a variety of implementation strategies.^38,39,40^ It convened a task force early during the study period; members included a primary care physician, a clinical social worker, and a psychiatrist, as well as clinic and health system leadership representatives. The task force met weekly initially and then monthly to organize clinician implementation team meetings, capture and share local knowledge, identify champions, and stage implementation scale-up. The task force assessed progress in depression screening by conducting audits and providing feedback with the help of data experts. They developed and organized quality-monitoring systems, reexamining the implementation and making adjustments as needed to address disparities identified during routine evaluation of screening rates (eg, by sex [female vs male], race and ethnicity, language, and age; in 2019, new- and established-patient annual health assessment forms were updated to include gender identity, but given an initial low response during the study period, only female and male sex are included in this analysis). Examples of adjustments included tailored strategies, such as making the questionnaires available in the most widely spoken non-English languages in the primary care practices; ongoing training that included retraining medical assistants in conducting depression screening; and, in December 2018, changing the recording system and facilitating the relay of clinical data to providers (eg, incorporating depression screening as a topic into the health system’s health care maintenance banner, a tab in the EHR, visible to everyone who opens a patient chart, where pending health care maintenance tasks appear for medical assistants and clinicians to address during clinical visits).

### Study Sample

We generated a data set from the EHR using specific criteria. Our cohort study included all eligible primary care patients aged 18 years and older who were seen from September 1, 2017, to December 31, 2019, in any of 6 UCSF Health adult primary care practice (excluding the seventh primary care practice, which serves primarily homebound older adults). We included patients if they had at least 1 outpatient primary care visit during the study period. We excluded patients with a diagnosis at baseline of depression, bipolar disorder, schizophrenia, schizoaffective disorder, or dementia and, for subsequent visits, after a new diagnosis of depression. For each year, we also excluded patients who were not screened that year but were not due for screening at any of their visits (eg, they had already been screened within 12 months prior to the visit). We defined depression as having an *International Statistical Classification of Diseases and Related Health Problems, Tenth Revision* depression diagnosis code between F32.0 and F34.1 listed in any encounter or on the problem list.

### Primary Outcome

The primary outcome, depression screening, was defined as completion of the Patient Health Questionnaire-2 (PHQ-2) during at least 1 patient visit during the specified study year. The PHQ-2 is a well-validated and widely used measure to screen for current depressive symptoms,^31,32,33,34^ including among racial and ethnic minority individuals and individuals with limited English proficiency.^41,42,43,44^ Medical assistants conducted depression screening either verbally or by giving patients a self-administered questionnaire, which was then entered manually in the EHR flow sheet. While patients could refuse to complete the PHQ-2, the EHR did not capture refusal; these instances were recorded as the PHQ-2 not completed. Patients with a positive result for depression on screening were asked to complete the full PHQ-9,^45^ the results of which then appeared as a decision support alert in the EHR for the visit clinician. The clinician could then arrange close follow-up; initiate a medication; or refer the patient to a behavioral health team, social worker, or psychiatrist as deemed appropriate.

### Definition of Covariates

Self-reported race and ethnicity and preferred language for health care were obtained from all new patients. The EHR did not contain information on the English proficiency of patients with a non-English preferred language. For this analysis, to compare English and non-English language preference groups and also assess depression screening by race and ethnicity within the English-speaking group, we created a single language-race-ethnicity variable with non–English language preference (ie, having a language barrier) and English language preference categories.

Additional demographic and patient characteristics included sex, age, baseline Elixhauser comorbidity count,^46,47^ and insurance type.

### Annual Screening Rates

For the rollout period (September 1, 2017, to December 31, 2017) and each subsequent calendar year (January 1 to December 31, 2018, and January 1 to December 31, 2019), we calculated a screening rate to reflect the percentage of all patients with a visit during that year who were screened for depression during at least 1 visit that year. We determined how many patients were screened out of all eligible patients seen within the time period. The denominator for each study time period (the 2017 rollout, 2018, and 2019) included all patients with a primary care visit during that time period in which they were eligible for depression screening. Some patients might have been eligible and seen each year, so an individual patient could contribute up to 3 data points.

### Statistical Analysis

We estimated overall rates of screening by patient demographic characteristics and by year in the study period using descriptive statistics. We evaluated the probability of patients being screened in 2018 and 2019 using logistic regression models for each year that included as predictors sex (female or male), language-race-ethnicity, age, comorbidities, health insurance type (private, Medicare, or Medi-Cal), and primary care site. Given that prior work in this health system found sex and language-race-ethnicity interactions in mental health care,^17^ we tested for associations between age and language-race-ethnicity group and sex and language-race-ethnicity group using Wald tests.

To determine whether associations among demographic variables and screening rates changed over time, we conducted a complementary analysis estimating overall rates of screening by yearly quarter during the study period. We combined data from all quarters of the study to model predictors of screening and tested for significant interactions among key covariates (sex, language-race-ethnicity, age, and health insurance type) and yearly quarter using Wald tests. The model accounted for multiple observations by patient across yearly quarters by adjusting for random patient effects. Statistical significance was defined as a 95% CI excluding 0 for differences and excluding 1 for ratios.

All tests were 2-tailed. We used Stata software, version 16.1 (StataCorp LLP), for all analyses.

## Results

There were 52 944 unique, eligible patients with 1 or more visits in 1 of the 6 primary care practices during the entire study period (59% female; mean [SD] age, 48.9 [17.6] years; 178 [0.3%] American Indian/Alaska Native, 13 241 [25.0%] English-speaking Asian, 3588 [6.8%] English-speaking Black/African American, 4744 [9.0%] English-speaking Latino/Latina/Latinx, 760 [1.4%] Pacific Islander, 22 689 [42.9%] English-speaking White, 4857 [9.0%] English-speaking other [including individuals who indicated race and ethnicity as other and individuals for whom race and ethnicity data were missing or unknown], and 2887 [5.5%] with language barriers [non–English language preference]). Table 1 shows the demographic characteristics of patients screened during the study period. During the rollout period (September 1, 2017, to December 31, 2017), 7551 (40.5%) of 18 642 eligible patients (95% CI, 39.8%-41.2%) were screened for depressive symptoms using the PHQ-2. In 2018, depression screening increased to 71.4% of eligible patients (24 684 of 34 555 patients; 95% CI, 71.0%-71.9%) and in 2019 increased to 88.8% of eligible patients (32 848 of 36 974 patients; 95% CI, 88.5%-89.1%).

**Table 1.** Patient Demographic Characteristics and Screening by Year^a^

Characteristic	Patients screened, No. (%)		
	Rollout (n = 7551)^b^	2018 (n = 24 684)	2019 (n = 32 848)
Positive depression screen	469 (6.2)	1343 (5.4)	2069 (6.3)
Sex^c^			
Female	4722 (41.1)	15 666 (74.4)	19 674 (89.4)
Male	2828 (39.5)	9016 (66.8)	13 168 (88.0)
Language-race-ethnicity group			
Non-English language preference			
Chinese	185 (24.1)	533 (51.7)	952 (86.9)
Spanish	61 (40.9)	155 (66.2)	201 (84.5)
Other^d^	138 (23.4)	433 (53.0)	694 (87.4)
English language preference			
American Indian/Alaska Native	26 (40.0)	94 (79.0)	112 (91.1)
Asian	2018 (43.4)	6450 (74.2)	8443 (89.5)
Black/African American	576 (37.5)	1739 (69.2)	2219 (89.3)
Latino/Latina/Latinx	733 (42.6)	2273 (73.7)	2882 (89.7)
Pacific Islander	102 (40.6)	348 (71.2)	465 (91.2)
White	3087 (41.6)	10 604 (72.4)	13 926 (88.3)
Other^e^	625 (41.6)	2055 (70.2)	2954 (88.9)
Age category, y			
18-30	969 (44.3)	3812 (75.9)	5360 (89.9)
31-44	1857 (44.7)	6827 (76.0)	8651 (89.1)
45-54	1269 (42.2)	4243 (72.8)	5321 (88.1)
55-64	1303 (38.8)	4188 (70.0)	5371 (88.2)
65-74	1207 (37.1)	3535 (66.6)	5021 (88.6)
≥75	946 (35.3)	2079 (60.5)	3124 (89.1)
No. of comorbidities, mean (SD) [range]	1.3 (1.5) [0-10]	1.1 (1.3) [0-10]	1.1 (1.3) [0-12]
Health insurance type			
Private	4193 (44.5)	15 402 (75.0)	20 190 (89.1)
Medicare	2304 (35.6)	6040 (64.4)	8618 (88.5)
Medi-Cal	900 (38.6)	2706 (69.6)	3484 (88.5)
Other (self-pay, VA, Workers’ Compensation)	154 (37.6)	536 (71.1)	556 (86.2)

Abbreviation: VA, Veterans Administration.

^a^ There were 18 642 patients in the rollout period (7551 [40.5%] screened); 34 555 in 2018 (24 684 [71.4%] screened, and 36 974 in 2019 (32 848 [88.8%] screened).

^b^ Rollout period was from September 1, 2017, to December 31, 2017.

^c^ In 2019, new and established patient annual health assessment forms were updated to include gender identity. However, given an initial low response during the study period, only female and male sex were included in this analysis.

^d^ Included all other languages spoken in the clinic; the next most common spoken languages in the health system were Vietnamese and Russian.

^e^ Other included individuals who indicated race and ethnicity as other and individuals for whom race and ethnicity data were missing or unknown.

In 2018, the first full year of depression screening, there were statistically significant differences in screening by sex, age, language-race-ethnicity group, and health insurance type (Table 1). Interactions between age and language-race-ethnicity group and sex and language-race-ethnicity group were not statistically significant and are not shown. Men had lower screening rates than women (adjusted odds ratio [aOR] 0.82, 95% CI, 0.78-0.86), and depression screening rates decreased with increasing age (aOR, 0.89 [95% CI, 0.82-0.98] for ages 45-54 and aOR, 0.75 [95% CI, 0.65-0.85] for ages 75 and older compared with ages 18-30) (Table 2). Patients with a Chinese language preference had lower odds of being screened for depression compared with English-speaking White patients (aOR, 0.59 [95% CI, 0.51-0.67]). Patients who preferred languages other than English, Chinese, or Spanish (including all other languages spoken in the clinic; the next most common spoken languages in the health system were Vietnamese and Russian) were the least likely to be screened for depression (aOR, 0.55; 95% CI, 0.47-0.64); however, screening was lower for all groups with non-English language preference compared with language-race-ethnicity groups with English language preference. Individuals with public insurance (aOR, 0.85 [95% CI, 0.78-0.93] for Medicare and aOR, 0.89 [95% CI, 0.82-0.96] for Medi-Cal) had lower screening rates than individuals with private insurance.

**Table 2.** Multivariable Logistic Regression Analysis of Depression Screening by Year

Variable	aOR (95% CI)^a^	
	2018	2019
Sex^b^		
Male	0.82 (0.78-0.86)	0.87 (0.81-0.93)
Female	1 Reference	1 Reference
Language-race-ethnicity group		
Non-English language preference		
Chinese	0.59 (0.51-0.67)	0.95 (0.79-1.15)
Spanish	0.94 (0.71-1.25)	0.75 (0.52-1.06)
Other^c^	0.55 (0.47-0.64)	0.98 (0.79-1.23)
English language preference		
American Indian/Alaska Native	0.95 (0.86-1.05)	1.37 (0.74-2.56)
Asian	1.04 (0.98-1.11)	1.17 (1.07-1.27)
Black/African American	1.51 (0.95-2.40)	1.15 (1.00-1.33)
Latino/Latina/Latinx	1.00 (0.92-1.11)	1.19 (1.05-1.35)
Pacific Islander	0.87 (0.71-1.07)	1.40 (1.03-1.92)
White	1 Reference	1 Reference
Other^d^	0.85 (0.78-0.93)	1.06 (0.94-1.20)
Age category, y		
18-30	1 Reference	1 Reference
31-44	1.01 (0.93-1.10)	0.92 (0.83-1.02)
45-54	0.89 (0.82-0.98)	0.84 (0.75-0.94)
55-64	0.86 (0.78-0.94)	0.88 (0.77-0.99)
65-74	0.85 (0.76-0.96)	0.95 (0.80-1.13)
≥75	0.75 (0.65-0.85)	0.98 (0.81-1.19)
No. of comorbidities, mean (SD)	0.96 (0.94-0.98)	1.00 (0.97-1.03)
Health insurance		
Private	1 Reference	1 Reference
Medicare	0.85 (0.78-0.93)	0.90 (0.79-1.03)
Medi-Cal	0.89 (0.82-0.96)	0.93 (0.83-1.04)
Other (self-pay, VA, Workers’ Compensation)	0.89 (0.75-1.05)	0.76 (0.60-0.95)

Abbreviations: aOR, adjusted odds ratio; VA, Veterans Administration.

^a^ All multivariable models are adjusted for primary care site. Associations between age and language-race-ethnicity group and sex and language-race-ethnicity group were not statistically significant and are not shown.

^b^ In 2019, new and established patient annual health assessment forms were updated to include gender identity. However, given an initial low response during the study period, only female and male sex were included in this analysis.

^c^ Included all other languages spoken in the clinic; the next most common spoken languages in the health system were Vietnamese and Russian.

^d^ Included individuals who indicated race and ethnicity as other and individuals for whom race and ethnicity data were missing or unknown.

In 2019, depression screening increased for all groups, regardless of sex, language-race-ethnicity group, age, or health insurance type (Table 1). In the multivariable logistic regression model (Table 2), men still had lower odds of being screened for depressive symptoms than women (aOR, 0.87, 95% CI, 0.81-0.93), although screening rates by sex were nearly identical by the end of 2019 (Figure). Odds of being screened were also higher, compared with English-speaking White patients, among Black/African American English speakers (aOR, 1.15; 95% CI, 1.00-1.33) and most other English-speaking racial and ethnic groups, including Asian (aOR, 1.17; 95% CI, 1.07-1.27), Latino/Latina/Latinx (aOR, 1.19; 95% CI, 1.05-1.35), and Pacific Islander patients (aOR, 1.40, 95% CI, 1.03-1.92). In 2019, screening among patients with non-English language preference was no longer significantly different than among patients with English preference. Similarly, screening among the oldest patients (aOR, 0.98; 95% CI, 0.81-1.19) and those with public health insurance (aOR, 0.90 [95% CI, 0.79-1.03] for Medicare and aOR, 0.93 [95% CI, 0.83-1.04] for Medi-Cal) was no longer significantly different than screening among the youngest patients and those with private insurance, respectively.

**Figure.**
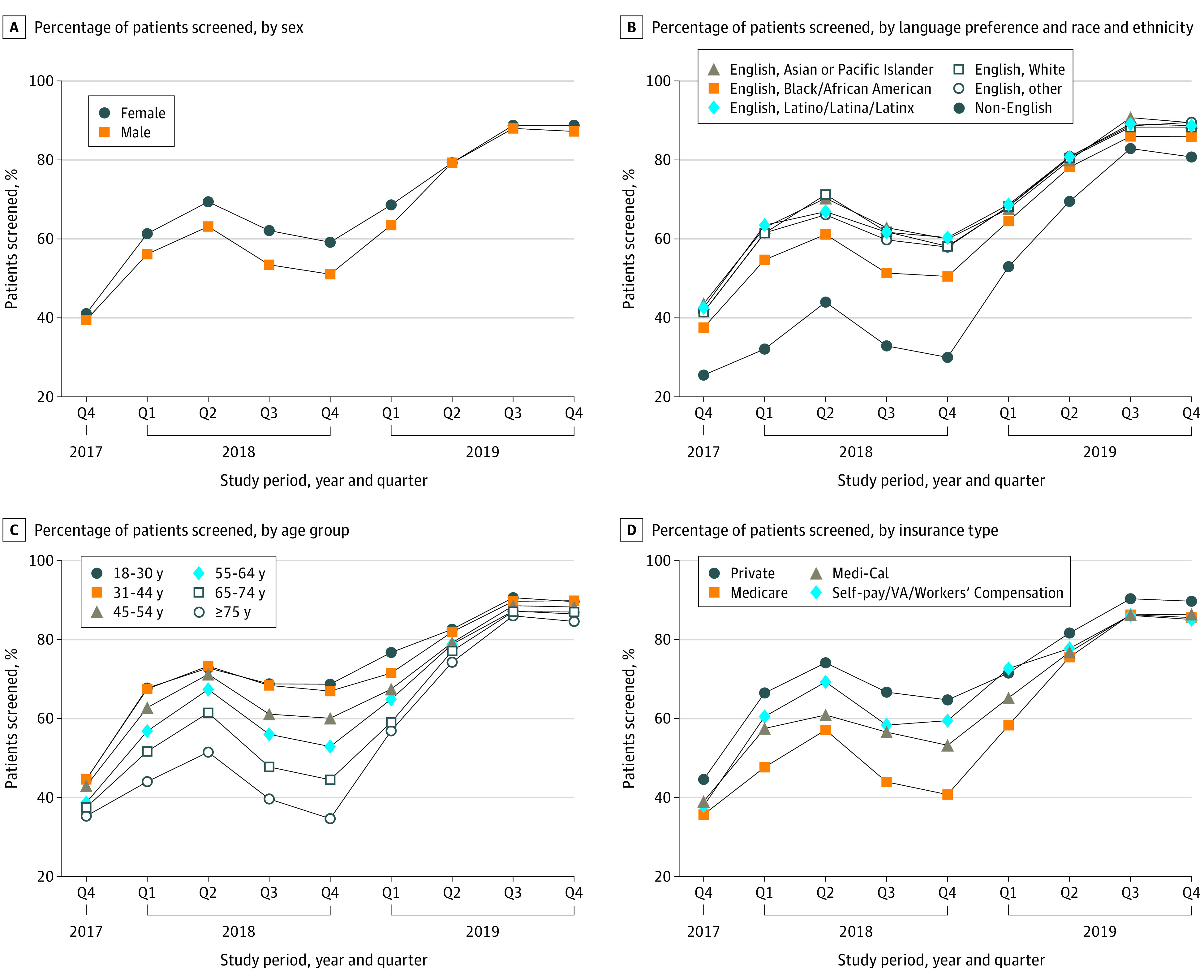
Depression Screening Rates During the Study Period (September 2017 to December 2019) by Patient Characteristics (Sex, Language-Race-Ethnicity, Age, and Insurance Type) A and C, *P* < .001; tests for significant associations among key covariates (sex, language-race-ethnicity group, age, and health insurance) were conducted to determine whether associations between demographic variables and screening rates changed over time. B, Other was defined as individuals who indicated race and ethnicity as other and individuals for whom race and ethnicity data were missing or unknown.

In our secondary analysis, for each group (sex, language-race-ethnicity, age group, and health insurance type), there was a steady decrease in screening differences over the course of 2019, resulting in resolution or near resolution of disparities by the last 2 quarters of 2019 (Figure).

## Discussion

In this cohort study, through implementation of adult depression screening in primary care, a large academic health system achieved high rates of depression screening among all patients, including groups at risk for underrecognition and undertreatment of depression. The substantial disparities in depression screening observed early in the rollout period, which were consistent with disparities reported in prior studies,^23,34^ were greatly reduced once screening was fully implemented in primary care. This was true for all groups at high risk for undertreatment of depression, including men, Black/African American individuals, those with English language preference, older patients, and those with public insurance.^23,34^ In addition, this health system accomplished much higher screening rates than previously published.^23,24,25,34^

Barriers to depression screening in primary care are multifactorial, including patient underreporting of symptoms, concerns about stigma, time pressures, and competing demands.^20,48,49,50,51,52,53^ In the current study, implementing a system-based screening program in which staff conducted screening in a routine manner, appears to have alleviated some of these barriers. Several factors may have contributed to achieving high, more equitable screening rates in this health system. First, during this time, depression screening was a priority for the system as part of a larger focus on quality improvement metrics implemented across safety net systems in California that are tied to state funds. Thus, the health system provided primary care practices with resources and support for implementation of this program. Second, from the launch of implementation, the health system convened a task force with representation from all primary care practices that was focused on identifying depression screening disparities in clinical settings and making adjustments. Third, we hypothesize that the inclusion of depression screening in the health care maintenance banner in 2018, which aligned depression screening seamlessly with established clinical workflows, likely contributed to increasing screening rates during 2019. Fourth, the availability of the screening tool in multiple languages and established access to professional interpreters in primary care may have facilitated implementation. Clinical staff in the primary care practices also speak multiple non-English languages, which may have further supported screening of patients with non-English language preference. This alignment of local quality improvement efforts, health system priorities, reimbursement policies, and a health equity lens for improvement efforts created a favorable environment to implement and improve adult depression screening in these primary care practices.

Depression screening is necessary, but not sufficient, to decrease depression care disparities.^34,54^ Screening may help with poor physician recognition of depressive symptoms, but screening must be followed by clinical action. Prior work has demonstrated that men,^55^ patients with language barriers,^22,35^ racial and ethnic minorities,^35,55,56^ and older patients^35,56^are less likely to receive adequate depression treatment.^57^ Yet effective depression treatment models exist, and these undertreated groups do achieve symptom remission when engaged in depression care.^10,12,13,58,59,60,61^ It is unclear whether improving equity in depression screening will translate into equal benefit from depression care. Future evaluations might center on whether screening is associated with increases in appropriate depression diagnosis; initial treatment; adequate follow-up; and, ultimately, remission.

### Limitations

This study has limitations. We relied on an EHR-based data set, which may limit the accuracy of race and ethnicity and preferred language data. Similarly, while gender minority groups disproportionately suffer from depression and its undertreatment,^62^ we were unable to assess screening rates among individuals in these groups because more inclusive gender identity data were not collected until 2019. Given the lack of data prior to implementation of the USPSTF guideline on depression screening, we are unable to characterize screening rates prior to this date. In fact, because we were not able to capture screening rates prior to depression screening rollout, our study may underestimate the true magnitude of the disparities reduction after depression screening implementation for groups at risk for underrecognition and undertreatment of depression. We were also unable to determine the quality of screening conducted; whether clinic conditions were conducive to administration of the PHQ-2 (eg, whether screening questions were asked or the screener questionnaire was completed independently in a quiet, confidential environment); or rates of patient refusal of screening, although, given the high rates of screening that were achieved by 2019, refusal may have been uncommon. Furthermore, the extent to which reduction in screening disparities led to similar reductions in disparities in actual treatment rates was outside the scope of this study. In the end, we can only hypothesize, based on knowledge of the health system rollout and implementation, what led to improvement in screening disparities by the end of the study period.

## Conclusions

In a large academic health system, depression screening substantially increased over time, and screening disparities substantially diminished after full implementation of adult depression screening in primary care. Given well-documented depression care disparities for men, racial and ethnic minority individuals, patients with language barriers, older patients, and patients with public insurance, a focus on implementing depression screening and initial depression treatment in primary care may help to improve depression recognition and appropriate treatment for all patients.
